# A Machine Learning Processing Pipeline for Reliable Hand Gesture Classification of FMG Signals with Stochastic Variance

**DOI:** 10.3390/s21041504

**Published:** 2021-02-22

**Authors:** Mohammed Asfour, Carlo Menon, Xianta Jiang

**Affiliations:** 1 Department of Computer Science, Memorial University of Newfoundland, St. John’s, NL A1B 3X5, Canada; masfour@mun.ca; 2 Biomedical and Mobile Health Technology Laboratory, Department of Health Sciences and Technology, ETH Zürich, 8008 Zürich, Switzerland or cmenon@sfu.ca; 3 Menrva Research Group, Schools of Mechatronic Systems Engineering and Engineering Science, Simon Fraser University, Metro Vancouver, BC V5A 1S6, Canada

**Keywords:** force myography, hand gestures recognition, machine learning, data pre-processing

## Abstract

ForceMyography (FMG) is an emerging competitor to surface ElectroMyography (sEMG) for hand gesture recognition. Most of the state-of-the-art research in this area explores different machine learning algorithms or feature engineering to improve hand gesture recognition performance. This paper proposes a novel signal processing pipeline employing a manifold learning method to produce a robust signal representation to boost hand gesture classifiers’ performance. We tested this approach on an FMG dataset collected from nine participants in 3 different data collection sessions with short delays between each. For each participant’s data, the proposed pipeline was applied, and then different classification algorithms were used to evaluate the effect of the pipeline compared to raw FMG signals in hand gesture classification. The results show that incorporating the proposed pipeline reduced variance within the same gesture data and notably maximized variance between different gestures, allowing improved robustness of hand gestures classification performance and consistency across time. On top of that, the pipeline improved the classification accuracy consistently regardless of different classifiers, gaining an average of 5% accuracy improvement.

## 1. Introduction

Hand gesture recognition has been widely applied in areas varying from simulated environments such as virtual reality (VR) integration [1] to real-world environments such as human-robot interaction [2,3] and prosthesis control [4]. Typically, by employing machine learning algorithms, hand gestures can be classified based on visual data from cameras [5], inertial data obtained from a gyroscope or accelerometer [6], or muscle activity data such as surface Electromyography (sEMG) [7,8]. Compared to visual-based and inertial-based methods, muscle activity-based hand gesture recognition does not have occlusion problems and can detect fine motor hand gestures and possibly gesture performing strength [9,10].

sEMG is the most established muscle activity-based hand gesture recognition technique [11,12] whose sensors are mounted on the upper limb to detect muscles’ electrical signals for gesture classification. sEMG signal processing and classification have been investigated thoroughly for a variety of applications [4,13,14,15,16]. Force myography (FMG) [17] is an emerging alternative technology that has attracted attention recently in hand gesture recognition [18,19]. FMG-based hand gesture recognition method utilizes an array of force-resisting sensors surrounding a specific part of the limb to capture the underlying musculotendinous complex’s volumetric changes during performing gestures [20]. Unlike sEMG, FMG has advantages that are robust to external electrical interference and sweating, inexpensive, and easy-to-use. By employing sophisticated machine learning techniques, FMG can achieve competitive performance as sEMG [21]. Jiang et al. [22] analyzed FMG sensors’ performance in hand gesture classification compared to that of sEMG. The results show that using as few as 8 FMG sensors for classification obtained accuracy as good as those of commercially available sEMG sensors in a controlled study.

However, similar to sEMG, FMG also has a downside caused by stochastic signal variation within the same class of gestures along time lasting, leading to low inter-session classification performance [23,24]. To overcome this problem, usually feature engineering and sophisticated machine learning algorithms are employed [25,26]. Tkach et al. [25] investigated the stochastic variance of sEMG signals on classification performance and how to overcome such an effect. They concluded that choosing robust features instead of raw sEMG signals could combat the effect; yet, they recognized that feature selection was not an adequate solution to eliminate such effect, and further research should be conducted to improve the robustness of sEMG signals classification. Ketykó et al. [26] inspected the variation of sEMG signals between sessions and between different participants, also known as domain shift. The shift affects the accuracy of models using such signals data. They proposed a recurrent neural network (RNN) to combat such an effect on sEMG and analyzed their model’s performance on public sEMG datasets. However, using half the trials, 50% of the data of CapgMyo dataset [27], without limitations on their order in training, could reduce the variance effect by itself. As described in the study, trials 1, 3, 5, 7, and 9 were chosen to train their classifier, reducing the variance effect compared with choosing trials 1, 2, 3, 4, and 5 for training.

This paper proposes a novel pre-processing pipeline to reduce the stochastic variance of FMG signals in hand gesture classification. Firstly, Fisher’s Discriminant Analysis (FDA) [28] clusters data points of the same class together while separating them from other classes’ data. Secondly, Principal Component Analysis (PCA) [29] eliminates correlation between data features. Lastly, Uniform Mapping and Approximation Projection (UMAP) [30] learns an internal pattern of the data that yields enhanced data features. To the best of our knowledge, this is the first work that utilizes FDA, PCA, and UMAP together as a data processing pipeline to improve hand gesture classification performance significantly using FMG. The proposed method’s performance and the processed dimensions robustness were evaluated using five different classifiers by comparing the classification results based on the pipeline processed data to those based on raw FMG data.

## 2. Materials and Methods

### 2.1. Pipeline Design

**Figure 1:**
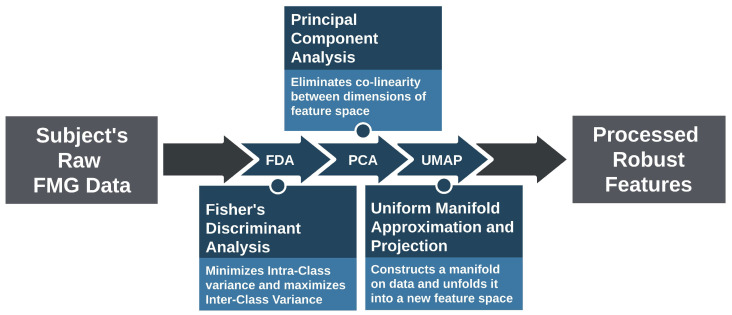
Schematic drawing of the pipeline design. FDA model minimizes the intra-class variance and maximizes the inter-class variance; PCA model removes correlation between data dimensions; UMAP model constructs a manifold on the data and unfolds it into a new feature space.

The pipeline consisted of three successive data processing models employing FDA, PCA, and UMAP algorithms, respectively, as shown in Figure 1.

**Figure 2:**
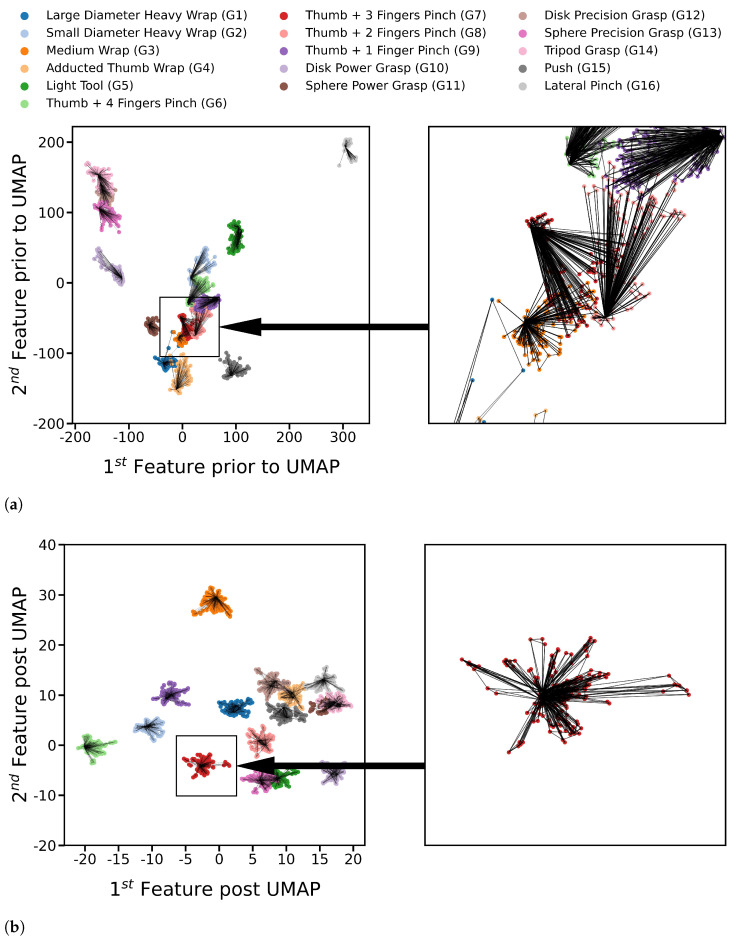
UMAP connections using tuned hyperparameters on participant two training data prior to and post applying UMAP, the final step of the pipeline (**a**) The connections of UMAP visualized on the first and second output features of FDA followed by PCA. The connections of gesture G7 points and their neighborhood are enlarged, showing the classes’ overlap. (**b**) The connections of UMAP visualized on the first and second final output features of the pipeline post applying UMAP. The connections of gesture G7 are enlarged, showing the class separability post applying UMAP.

- **FDA Model:** The raw FMG data first underwent Fisher’s Discriminant Analysis (FDA) [28] processing to maximize class separation linearly, reduce the degree of overlap between different classes, and thus improve the overall effect of the pipeline. FDA is a processing technique that finds the linear boundary separating different classes’ data. This boundary maximizes the distances between different classes’ points, inter-class variance, while minimizing distances between the same class points, intra-class variance. FDA solves a constrained optimization function using Lagrangian multipliers
  (1) $$L\left(\lambda ,W\right)=trace\left(W^{T}\;S_{B}\;W\right)\;-\;\lambda \left(trace(W^{T}\;S_{W}\;W)\;-\;1\right)$$
  where $S_{B}$ is the variance between classes, inter-class variance, and $S_{W}$ is the variance within each of the classes, intra-class variance. Both of the variances matrices’ dimensions are d*d where *d* is the number of the original data dimensions. The solution *W*, containing the basis vectors of the transformation space, is given by
  (2) $$W=eigen(S_{W}^{-1}\;S_{B})$$
- **PCA Model:** After FDA processing, PCA [29] was applied to remove the correlation or dependence between data dimensions. The elimination of correlation benefited the final step of the pipeline, UMAP, significantly, as it learned the underlying structure more easily given no dimensions’ correlation or relationship needed to be taken into account. The principal components can be obtained from
  (3) $$U=eigen(\frac{1}{n}\;{\left(X-\mu \right)}^{T}\;\left(X-\mu \right))$$
  where $X_{n*d}$ is the matrix of *n* data samples in *d*-dimensional space, and $\mu_{1*d}$ is the mean of the data.
- **UMAP Model:** Finally, UMAP [30] was used to produce a more robust set of data features. UMAP assumes the data is distributed on a connected manifold, a nonlinear surface, that resembles a new Euclidean space if unfolded. UMAP connects data points to construct that manifold, followed by an optimization step to find the transformation yielding the data points’ representation on the unfolded manifold features. UMAP optimization is weighted between conserving the relative positions of points through the established connections and separating different classes via the points’ labels. The graph resulting from UMAP connections between training points for participant two after applying FDA and PCA is depicted in Figure 2a, whereas the same points distribution with the same connections after applying UMAP is shown in Figure 2b. UMAP [31,32] algorithm has many hyperparameters to tune its manifold. One manifold can preserve the original structure of the data, whereas another separates the classes non-linearly. Finding the best transformation to separate different classes’ points was preferred due to the stochastic variance of FMG signals, and participant two’s data was used for tuning. The separation’s precedence was considered during the tuning of hyperparameters listed in Table 1.

FDA is linear, and the transformation is based on gesture classes, while PCA is also linear but class agnostic. Both can be considered as pre-processing steps for UMAP, which is the vital step in the pipeline, yielding a consistent set of features throughout time sessions, yet its performance is further enhanced by eliminating certain artifacts beforehand using FDA and PCA. Even though UMAP finds a manifold that separates the classes, separating the classes linearly by the FDA has made UMAP’s optimization easier and has enhanced the set of possible UMAP manifolds. UMAP optimization would have been a more challenging problem without an initial linear separation, as it would have been more complicated to separate the overlapping classes non-linearly and harder to tune using the hyperparameters. By reducing the overlap using FDA, UMAP manifolds are able to give better results, as will be shown by the results.

PCA is used in the pipeline for correlation elimination. No dimensions were removed as it was found that UMAP’s chosen manifold, given a non-correlated set of features, separates the classes more consistently throughout time. It is notable to mention that PCA has been coupled with UMAP [31,32] in the literature for different purposes, such as data analysis and visualization, than that of the proposed pipeline.

### 2.2. Classification Models for Evaluation

Five machine learning algorithms were employed to evaluate the performance improvement after applying the data processing pipeline, compared to using raw FMG data as input to the classifiers [22,33]. The models used in this study are listed below.

- Linear Discriminant Analysis (LDA) [34] has been widely used in gesture recognition using FMG [17,35,36,37]. LDA assumes that classes are normally distributed in the feature space with the same variance for all classes.
- Quadratic Discriminant Analysis (QDA) [38] is similar to LDA, as it assumes classes are normally-distributed but with independent variances, giving a quadratic decision boundary.
- Support Vector Machine with Radial Basis Kernel (SVM-RBF) [39] uses a kernel to transform data into another feature space before finding a linear decision boundary in that new space. The radial basis function transforms data into infinite-dimensional space, theoretically. The linear decision boundary in that space is nonlinear in the original feature space.
- Fully-Connected Neural Network (FC-NN) [40] is the most complex of the used models. Only a few fully-connected layers were sufficient for this study. Neural networks have numerous hyperparameters to tune and random weights initialization, making them extremely flexible during design. One neural network architecture with fixed hyperparameters values was used to evaluate the processing effect subjectively. The hyperparameters used for it are listed in Table 2. Regularization techniques are used with the neural network for a better generalization of test data.
- K-nearest Neighbors (KNN) [41] is one of the most basic classifiers, which uses neighboring labeled data points to classify unlabelled data. The pipeline’s results for KNN with five neighbor points can be extended to most metric-dependent methods in unsupervised learning like clustering since these methods share the same principles.

The decision boundaries of these models have different orders of non-linearity. For instance, LDA has linear hyperplanes, FC-NN has highly nonlinear hypersurfaces, whereas KNN uses a distance metric instead of decision boundaries. The higher the classifier’s non-linearity or flexibility, the higher its learning capacity or complexity is said to be. By comparing different models with varying orders of non-linearity, the performances post applying the pipeline were studied thoroughly [42,43,44]. In other words, enhancement in all classifiers performance and similar accuracy results would suggest that the pipeline obtains a more robust feature space regardless of the classifier to be used.

An instance of the pipeline and an instance of each of the classifiers were trained and tested for each participant. For a single participant, the FMG data from the training session was used to calibrate the pipeline, such that the raw FMG training session data was used to fit the FDA model, which transformed the data to be used to fit the PCA model to transform the data once more on which UMAP was fitted. Then test sessions’ data went through the calibrated pipeline to derive the desired features. The training session data transformed by the pipeline was used to train the classifiers, whereas the test sessions’ data was transformed by the pipeline then used to evaluate each classifier’s accuracy.

### 2.3. FMG Data

The data used in this investigation was obtained from a previous study [33] investigating the force exertion effect on hand gesture classification. The data was collected using an array of 16 FMG sensors, specifically force-sensitive resistor (FSR), in a custom-designed wristband. The whole data collection process was divided into three sessions, a training session and two testing sessions. A total of 16 grasp gestures were performed in each session. The grasps were large diameter heavy wrap (G1), small diameter heavy wrap (G2), medium wrap (G3), adducted thumb wrap (G4), light tool (G5), thumb + 4 fingers pinch (G6), thumb + 3 fingers pinch (G7), thumb + 2 fingers pinch (G8), thumb + 1 finger pinch (G9), disk power grasp G(10), sphere power grasp (G11), disk precision grasp (G12), sphere precision grasp (G13), tripod grasp (G14), push (G15), and lateral pinch (G16).

In that study, nine participants, seven males and two females with a median age of (27 ± 6) years, were recruited. All were right-handed and self-reported to be 100% functional with their working hands with an average wrist size (17.3 ± 1.4) cm. All participants read and signed the consent form before the study, approved by Simon Fraser University. Data were collected from the participants throughout 180 trials. Each participant performed four trials for training and eight trials for each of the two testing sessions. All 16 channels of FSR signals were included in the data analysis with a sampling rate of 15 Hz [33].

Four repetitions for each grasp were performed in the training session using natural grasping force, which is similar to naturally grasping an object in daily life. Each repetition lasted 3 s, yielding 45 samples. Thus the 16 gestures with four repetitions each, lasting 12 s per gesture, yielded a total of 2880 training samples for each participant at the sampling rate of 15 Hz.

Alternatively, the testing sessions were conducted with eight repetitions of each gesture. Each of which was performed using eight different grasping force levels purposely, yielding 5760 test samples for each testing session for each participant, thus 11,520 samples for all 16 grasp types of each participant. None of the participants reported fatigue during the data collection.

A monitor guided the participants as it displayed the object’s image with the required exerted force level as a reference to match their force level on the monitor. There was only a short break between the sessions, and the band was not removed between different sessions. This data collection protocol was used for 9 participants to collect 16 gestures’ data with eight varying force levels for each gesture. For a detailed description of the data collection protocol, please refer to [33].

### 2.4. Statistical Analysis

Three-way ANOVA was computed to show the impact of different factors on the classification accuracy. The classifier type, the test session order, and the data feature space (raw FMG vs. pipeline features) were the three independent variables. Meanwhile, the classification accuracy was selected as the dependent variable. Post-hoc pairwise comparisons, Tukey’s HSD (honestly significant difference), were further conducted if there were any significant effects of the independent variables. The significance level was set to *p*-value = 0.05.

## 3. Results

**Figure 3:**
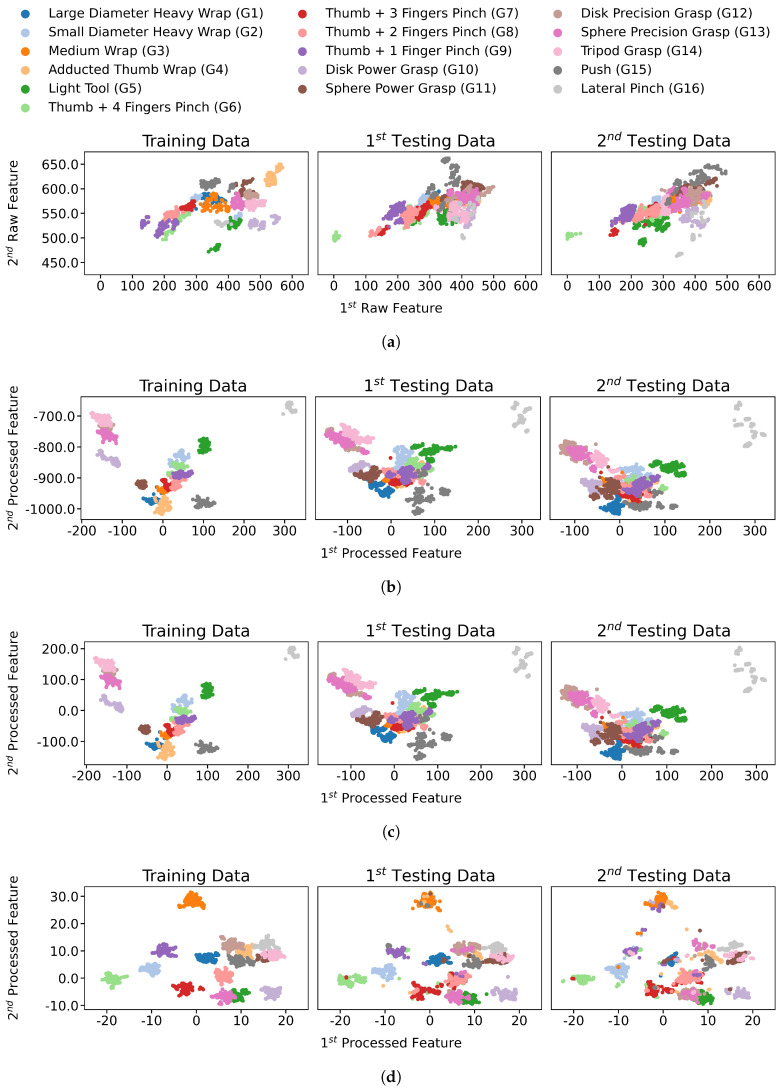
Feature spaces comparison between before- and after- applying each pipeline possessing step for all participant 2 data gestures, using the first two dimensions for each feature space. (**a**) Sessions data using raw features. (**b**) Sessions data post applying FDA, the first step of the pipeline. (**c**) Sessions data post applying PCA, the second step of the pipeline. (**d**) Sessions data post applying UMAP, the final step of the pipeline.

**Figure 4:**
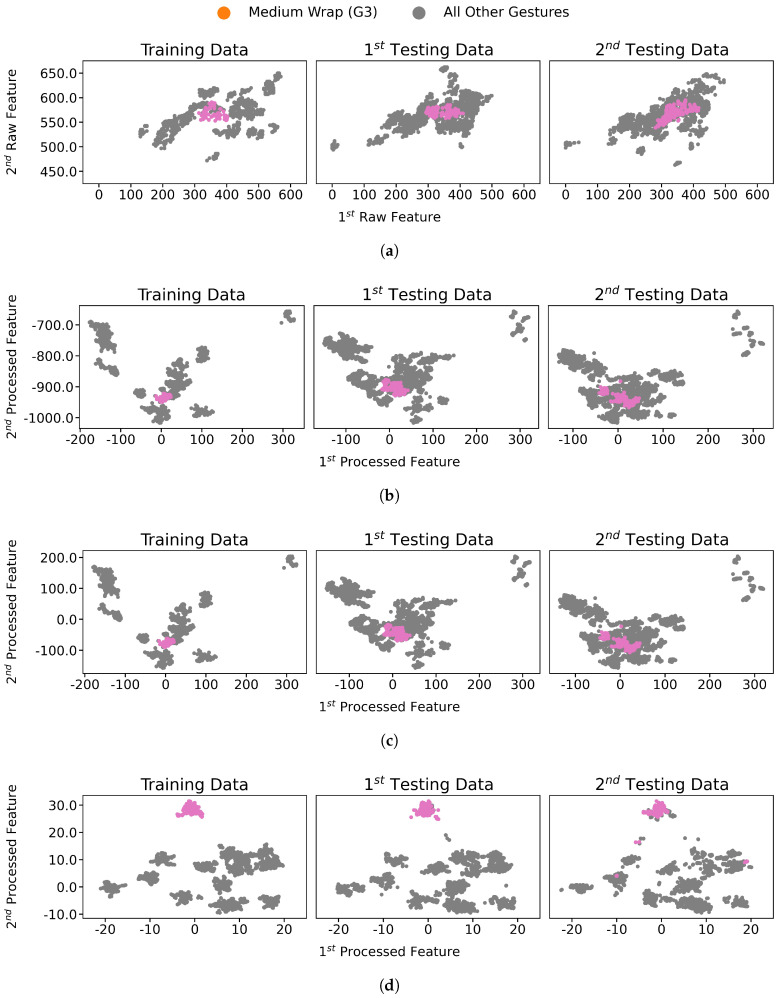
Feature spaces comparison of a single gesture between before- and after- each pipeline processing step, highlighting only medium wrap gesture (G3) of participant 2 training data, using the first two dimensions, the most dominant two, for each feature space. (**a**) Sessions data (G3) using raw features. (**b**) Sessions data (G3) post applying FDA, the first pipeline step. (**c**) Sessions data (G3) ost-applying PCA, the second pipeline step. (**d**) Sessions data (G3) post-applying UMAP, the final pipeline step.

**Figure 5:**
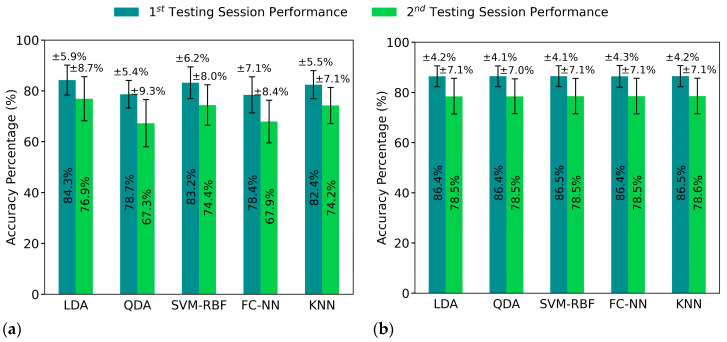
Classifiers’ mean accuracy over all participants for each test session using raw and pipeline features. The error bars are for one standard deviation. (**a**) Mean accuracy using the raw features as classifiers’ input. (**b**) Mean accuracy using the pipeline features as classifiers’ input.

**Figure 6:**
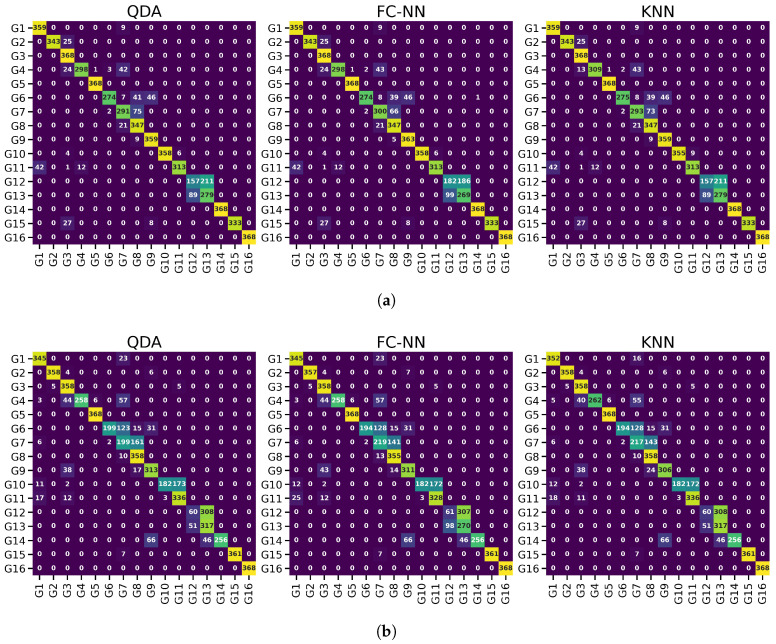
Confusion matrices for QDA, FC-NN, and KNN displaying per-class accuracy on the pipeline’s features using participant two’s both test sessions. (**a**) Confusion matrices using participant two’s first test session. (**b**) Confusion matrices using participant two’s second test session.

**Figure 7:**
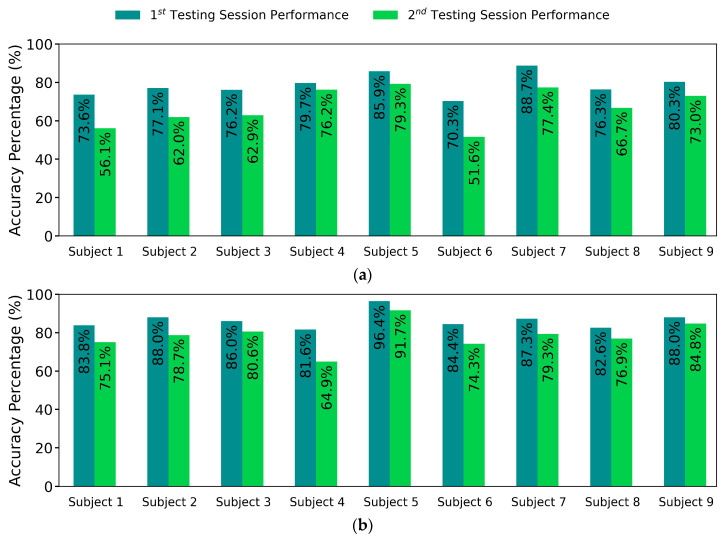
Comparison of QDA accuracy using raw and pipeline feature spaces per test session of each participant. (**a**) QDA accuracy results on raw features. (**b**) QDA accuracy results on the pipeline features.

The proposed pipeline improved the feature separation between classes and reduced the variation within each class; thus, the classification performance was significantly improved on the pipeline-processed features. The distribution transformation throughout each step of the pipeline is visualized in Figure 3 and focusing and one gesture distribution in Figure 4. The mean accuracy results are reported in Figure 5, whereas per-class accuracy for several classifiers is depicted in confusion matrices in Figure 6. Furthermore, QDA performance is investigated for all participants in Figure 7.

Figure 3 shows the comparison of exampling data distribution of participant 2 between the raw data (Figure 3a) and the data processed by the pipeline (Figure 3d), throughout the training and two testing sessions. In each plot, the x-axis and y-axis show the first two dimensions of either the raw FMG data or the pipeline’s features. The data distribution is well improved to be more distinguishable between classes after the pipeline processing (Figure 3d) compared to raw FMG data in Figure 3a. Specifically, the signal separation between classes, the inter-class variance, is improved, and the signal variance within a class, the intra-class variance, is reduced.

In order to show better reduction of the intra-class variation, Figure 4 highlights only a single gesture of participant 2 data while processing the data through the pipeline in the same manner as in Figure 3. Comparing Figure 4d to Figure 4a, we can see that the intra-class variance was reduced and the data points for each was more clustered after pipeline processing.

Figure 5 shows the mean accuracies over all participants for each of the test sessions using raw features (Figure 5a) and pipeline features (Figure 5b). We can see that the accuracies are consistently improved throughout different classifiers after pipeline processing for both testing sessions.

To further analyze the similarity of accuracy results between different classifiers, the per-class accuracies are obtained through confusion matrices in Figure 6. The confusion matrices are computed for 3 of the classifiers for participant two’s first and second test sessions.

The confusion matrices for participant 2 in Figure 6 further support the similarity of results. For each of the test sessions in the figure, three different classifiers misclassify almost the same overlapping outliers that are hard to classify correctly, regardless of the model employed.

Among the five classifiers, the QDA classifier is the one most benefited from applying the proposed pipeline to the data. Figure 7 shows each participant’s QDA accuracy results for raw features and the proposed pipeline features, respectively. As shown in Figure 7, the pipeline improves QDA performance for all participants, except for participant 4, where the second test session decreases its accuracy, which could be an outlier.

The stochastic variance of FMG signals led to declined classification performance and huge variance in accuracy of different classifiers (Figure 5a) and different participants (Figure 7a). The proposed pipeline reduced the aforementioned variance as it equalized the performances of different classifiers (Figure 5b), made the difference in the test sessions’ accuracy near-constant and reduced the variance for all classifiers results for the nine participants (Figure 7b).

To investigate individual steps’ impact on classification accuracy, results were obtained after applying each step of the pipeline individually in Table 3. These results, alongside the depiction of the transformation distribution at each step in Figure 3, would better explain the individual effects.

ANOVA results showed both the session order ( *F*${}_{1,199}$ = 59.304, *p* < 0.0001) and data feature space ( *F*${}_{1,199}$ = 29.690, *p* < 0.0001) impact the results significantly; whereas the classifier type had marginal significant effect to the classification accuracy ( *F*${}_{4,199}$ = 2.373, *p* = 0.054). There are no significant interactions between the independent variables, neither 2-way nor 3-way interactions. As the classifier type effect was marginally significant, we further applied 1-way ANOVA to the results based on raw FMG and pipeline processed features respectively, taking only classifier type as independent factor.

The results showed that there was a significant effect in classifier type to raw FMG based classification accuracy ( *F*${}_{4,99}$ = 3.083, *p* < 0.05), but there was no significant difference in the resulting accuracy between classifiers after pipeline pre-processing. Further post-hoc analysis on the raw FMG based classification showed LDA had significant higher accuracy than QDA (*p* < 0.05) and FC-NN (*p* < 0.01) respectively, affirming the results in Figure 5a. No other pairs of classifiers had a significant difference.

## 4. Discussion

### 4.1. Classification Performance Improvement

The classification accuracy was improved significantly and consistently throughout all the tested classifiers for both test sessions after applying the pipeline processing, as shown in Figure 5b. With varying complexity from simple to elaborate and linear to non-linear classifiers, all the five models experienced almost identical accuracy results with similar standard deviations of performances, regardless of various original performances before pipeline processing (Figure 5a). This illustrated that the models learned from the same underlying structure and that reliable features were produced by the proposed pipeline. This proved that the proposed pipeline produced consistent and reliable distribution features of the data given and enhanced all classification models’ performances by varying degrees.

The implied significantly separable distribution of pipeline processed features, compared to raw FMG features distribution, was supported by Figure 3d; meanwhile, the misclassifications using the processed features were due to noise in the data from different classes’ remaining overlap. The separable distribution of processed features needed only the simplest of decision boundaries to obtain the highest accuracy possible in the presence of noise; hence the classification accuracy became independent of the classifier’s complexity.

Without pipeline pre-processing, besides lower accuracies, there was significant variance in classification results between different classifiers, as shown in Table 3. A more interesting observation is that simpler models such as LDA achieved better accuracy using FDA and PCA features than higher complexity ones, such as FC-NN, the most complex model.

While the use of FDA or PCA features yielded results that were strongly dependent on the type of classifier (see Table 3), UMAP features appeared to be consistent and robust concerning the choice of the classifier, but unfortunately yielded worse mean accuracy results. Thus, the pipeline took advantage of both and enabled classifiers to achieve high classification accuracy while maintaining the robustness of accuracy across all classifiers. The comparison in Table 3 showcases the pipeline impact on the classification accuracy that could not be obtained through one of the methods; thus, supporting the pipeline’s concept of tackling the FMG variance characteristics sequentially in a combined manner gives more robust features.

The pipeline effect on all participants and enhancement of classifiers’ accuracy, despite their gestures distributions, shows the benefit of incorporating it into force myography applications. This is seen in the consistent results for different classifiers with a reduced deviation for different participants, which is beneficial in hand gesture applications that require prolonged FMG signal acquisition. The pipeline obtains a mean accuracy over all participants and classifiers of 86.4% with ±8.6% standard deviation for the first test session, and 78.5% with ±11.0% standard deviation for the second test session. The pipeline results surpass all its individual processing steps results in Table 3 while simultaneously reducing the variation in the accuracy results.

The classification accuracy using LDA based on raw FMG features in the present study was similar to the results from our previous study based on the same data set [33]. In contrast, the pipeline proposed in this study achieved more robust performance across different classification algorithms. Another similar state-of-the-art study was conducted by Anvaripour et al. [45], where almost 93% classification accuracy was achieved on six hand gestures using a similar FMG band on the forearm, and an advanced feature extraction technique was employed to enhance the performance of the classifier. However, considering the hand gesture number (16 grasps) used in our study, the performance achieved by our pipeline method is competitive to theirs. Furthermore, our testing condition was more rigorous, i.e., training the models using natural grasping force and testing two sessions gestures performed using eight different levels of grasping forces.

It may be worth pointing out that the pipeline could not eliminate the accuracy decline caused by the stochastic variance of FMG signals entirely. This can be observed from both Figure 5a,b, by comparing the accuracy of test session 1 to test session 2; there were still significant differences between the two testing sessions even after pipeline processing (Figure 5b). However, both testing sessions were improved significantly, demonstrating that the pipeline method works throughout time sessions.

### 4.2. Processed Distributions Similarity

The stochastic FMG variance produced different distributions for different time sessions. In Figure 3, participant two’s data is shown after each step of the pipeline. FDA managed to reduce the overlap to some extent, as seen in Figure 3b. Afterward, PCA removed the colinearity between dimensions. PCA can be seen as a combination of a translation and a rotation of the distribution. Even though only the first two dimensions of the distribution are shown in Figure 3c, PCA was not used for data reduction here and hence rotated the whole feature space.

Finally, UMAP in Figure 3d utilized these uncorrelated dimensions previously processed by both FDA and PCA to obtain the final output distribution of the pipeline and separate the classes significantly compared to the original distribution in Figure 3a.

The proposed pipeline increased the similarity of gesture distributions between the training session and the test sessions. Moreover, the pipeline separated the data and increased the inter-class variance, making the outliers in test sessions easier to be distinguished from the spread data points. In contrast, in Figure 3a, data distribution varied significantly from testing session 1 to testing session 2.

The reduction of intra-class variance is easier to be seen in Figure 4a with highlighted gesture (G3) data only, while Figure 4d illustrates gesture (G3) distribution on the pipeline’s features. FDA and PCA processing in Figure 4b,c are not sufficient to obtain the same degree of separation, as the (G3) gesture data still suffers from considerable inter-class variance and overlap, whereas the same gesture is entirely separated using the entire pipeline as shown in Figure 4d. The change in data distributions in Figure 4a is known as covariate shift [45]. The covariate shift is a general label from machine learning literature for the inter-session variance mentioned in this study. The figure shows that the distribution for gesture (G3) has a similar mean and variance through different sessions. Notably, there were still class outliers; however, most points had the same distribution across sessions. On the other hand, the gesture (G3) had a much higher intra-variance on raw features and overlapped with other gestures making its classification harder.

## 5. Limitation and Future Work

The pipeline method was tested on an FMG dataset from a study constrained to a lab setting, where the data collection was conducted in different time sessions with short breaks without taking off the FSR wristband during the break. Obtaining FMG data for a longer time would require wearing the FSR wristband for the entirety of the data collection protocol, as different wearings of the FSR wristband for different sessions would introduce sensor misalignment as a source of variance. Future research investigating the processing pipeline effect for more extended periods would be possible if the FMG data were collected during different wristband wearings while eliminating the variance due to sensors’ positions shift between sessions.

This pipeline method has only been tested with FMG data. Future work would extend the pipeline process to more muscle activity-based signals, such as sEMG, and also apply to more general classification problems without limited to hand gesture recognition.

The feature space obtained by the processing pipeline was not interpreted into human-understood features. The interpretability could help understand the correlation between specific muscle activations’ latent features and their corresponding hand gestures. For instance, the data of gestures (G5) and (G13) may seem unrelated according to FMG signals in Figure 3a, whereas the pipeline finds these two gestures very similar while clustering the data of each class in Figure 3d according to its processed features. Future investigation of the correlation between muscle group activation, corresponding to specific FMG sensors, and values of each dimension might yield a relationship between the processed dimensions and muscle activities that are human-understandable.

The proposed pipeline’s output signal has several preferred qualities, such as class separation and variance reduction within a class. These properties allow the pipeline to be potentially utilized in other applications like FMG signals analysis and interpretation and latent patterns discovery that are more robust to undesired effects such as the stochastic variance or overlap of different gestures’ data. Finally, this pipe method also has the potential to be utilized to enhance clustering techniques, given its class separability, as proved by the improvement in the KNN classifier, which uses similar concepts to several clustering techniques.

## 6. Conclusions

The present work proposed a data processing pipeline to improve hand gesture recognition performance using machine learning by producing consistent data features for different time sessions. The results showed that the pipeline effectively maximized the inter-class signal variance and minimized the within-class variance, separating different classes into unique clusters, thus improving the classification reliability and accuracy when using different classifiers. The pipeline performance was not affected by the participant’s data distribution and classifiers’ types, as shown in this study, where all participants’ and classifiers’ results were improved. This study’s findings help reduce hand gesture recognition variation due to muscle activity and FMG signals’ stochastic variance, and could have great potential to be applied in more general muscle activity-based classification problems.

Results support that the pipeline effect is not merely the robustness of the outputted features but also the linear separation of most data points classes; thus, allowing low complexity, easier to train, classifiers to achieve accurate results equal to that of much higher complexity classifiers. The similar performance of classification methods could enhance inference experience for real-time hand gesture classification of FMG signals. Compared to each of the steps the pipeline components were applied individually, the enhanced effect of adding them together as a whole pipeline demonstrated superiority to obtain robust and higher accuracy results of any individual step by deliberately tackling particular characteristics in the data, making the classification process more robust for different time sessions.

## Figures and Tables

**Table 1:** UMAP tuned hyperparameters in the pipeline.

UMAP Hyperparameter	Chosen Value
Number of Neighbors	2
Metric	Cosine Distance
Output Metric	Euclidean
Target Metric	Euclidean
Target Weight	0.75
Repulsion Weight	3.0
Embedding Initialization	Random
Minimum Distance	0.25

**Table 2:** Neural Network used hyperparameters.

Hyperparameter	Used Value
Learning Rate	0.001
Epochs	30
Batch Size	1024
Validation Split	0.2

**Table 3:** Mean accuracy results’ comparison between the pipeline and different features obtained by applying FDA, PCA, and UMAP individually, not sequentially. Classifiers’ results are obtained as a mean of both test sessions of all participants and rounded to 1 decimal place.

Classifier	FDA Features		PCA Features		UMAP Features		Pipeline Features	
	Session 1	Session 2	Session 1	Session 2	Session 1	Session 2	Session 1	Session 2
LDA	86.5%	77.4%	84.3%	76.9%	81.5%	72.5%	86.4%	78.5%
QDA	79.4%	67.7%	78.8%	67.3%	81.5%	72.5%	86.4%	78.5%
SVM-RBF	83.2%	75.4%	85.3%	76.0%	81.5%	72.5%	86.5%	78.5%
FC-NN	82.1%	72.8%	79.6%	68.4%	81.5%	72.6%	86.4%	78.5%
KNN	86.5%	78.4%	82.4%	74.2%	81.6%	72.5%	86.5%	78.6%
Mean	83.6%	74.2%	82.3%	72.5%	81.5%	72.5%	86.4%	78.5%
Std Dev	±10.4%	±12.9%	±11.4%	±13.8%	±10.8%	±12.1%	±8.6%	±11.0%

## Data Availability

Restrictions apply to the availability of these data. Data were obtained from the previous study “Force Exertion Affects Grasp Classification Using Force Myography. IEEE Trans. -Hum. -Mach. Syst. 2018” and are available from the authors with the permission of Jiang, X.; Merhi, L.; Menon, C.
